# Dichotomic response to interleukin-6 blockade in idiopathic multicentric Castleman disease: two case reports

**DOI:** 10.1186/s13256-021-02726-4

**Published:** 2021-03-07

**Authors:** Simone Ferrero, Simone Ragaini

**Affiliations:** 1 Division of Hematology, AOU “Città della Salute e della Scienza di Torino”, Torino, Italy; 2 Department of Molecular Biotechnologies and Health Sciences, University of Torino, Via Genova 3, 10126 Torino, Italy

**Keywords:** Idiopathic, Multicentric, Castleman, iMCD, IL-6, Blockade, Siltuximab, Case, Report

## Abstract

**Background:**

Human herpervirus-8/human immunodeficiency virus negative Idiopathic multicentric Castleman disease (iMCD) is a lymphoproliferative disorder sustained by a pro-inflammatory condition of hypercytokinemia mostly mediated by Interleukin-6 (IL-6). According to iMCD consensus guidelines, anti-IL-6 blockade should be the first-line therapy for iMCD. However, despite the existing therapeutic alternatives, a large proportion of iMCD patients still lacks an effective therapy.

**Case presentation:**

Here, we report two real-life iMCD cases with a different response to IL-6 blockade. The first presented patient obtained a prompt resolution of symptoms and a complete regression of adenopathies after IL-6 blockade therapy administration. Conversely, the second patient did not respond neither to *Rituximab* and *Etoposide* association nor to IL-6 blockade therapy (both *Siltuximab* and *Tocilizumab*). Furthermore, *Intravenous immunoglobulin*, *Cyclosporine A, Sirolimus* and anti-Interleukin-1 *Anakinra* were all attempted without any results. Since no treatment was successful, after a further confirmation of iMCD diagnosis by a second lymph node biopsy, patient has been candidate for thalidomide, cyclophosphamide and prednisone association therapy.

**Conclusions:**

The iMCD cases we reported are coherent with the evidences that IL-6 blockade is a safe and an effective therapy for iMCD. Despite this, more than half of patients do not respond to anti IL-6 drugs. In such cases, therapeutic alternatives could be represented by Sirolimus, targeting PI3K/AKT/mTOR signaling or by associations of conventional drugs such as thalidomide, cyclophosphamide and prednisone. However, the two reported iMCD cases, confirm the need to more deeply investigate iMCD pathogenesis and to better dissect the heterogeneity of the disease in order to develop novel, effective therapeutic strategies.

## Background

Castleman disease (CD) consists of an uncommon series of lymphoproliferative disorders comprehending several subsets. Among them, human herpervirus-8/human immunodeficiency virus (HHV-8/HIV) negative Idiopathic multicentric Castleman disease (iMCD) still remains one of the less properly understood subset(1). iMCD onset may include manifestations such as multiple lymphadenopathies, hepatosplenomegaly, cytopenias, and organ dysfunction. Furthermore, iMCD is classified into two categories (nonsevere or severe) according to Castleman disease collaborative network (CDCN) severity criteria based on patient performance status and organ dysfunction including renal disfunction, anasarca, severe anemia, and pulmonary involvement. Published guidelines (1) indicate the association of anti-interleukin-6 *Siltuximab* and steroids as first-line therapy for iMCD. In *Siltuximab* refractory patients, *Tocilizumab* and *Rituximab* are indicated, respectively, as second- and third-line therapy. Nonetheless, a large proportion of patients still do not respond to therapies. Here, we report two real-life iMCD cases, with different presentation, clinical course and response to *Siltuximab*.

## Case n. 1 presentation

**Fig. 1:**
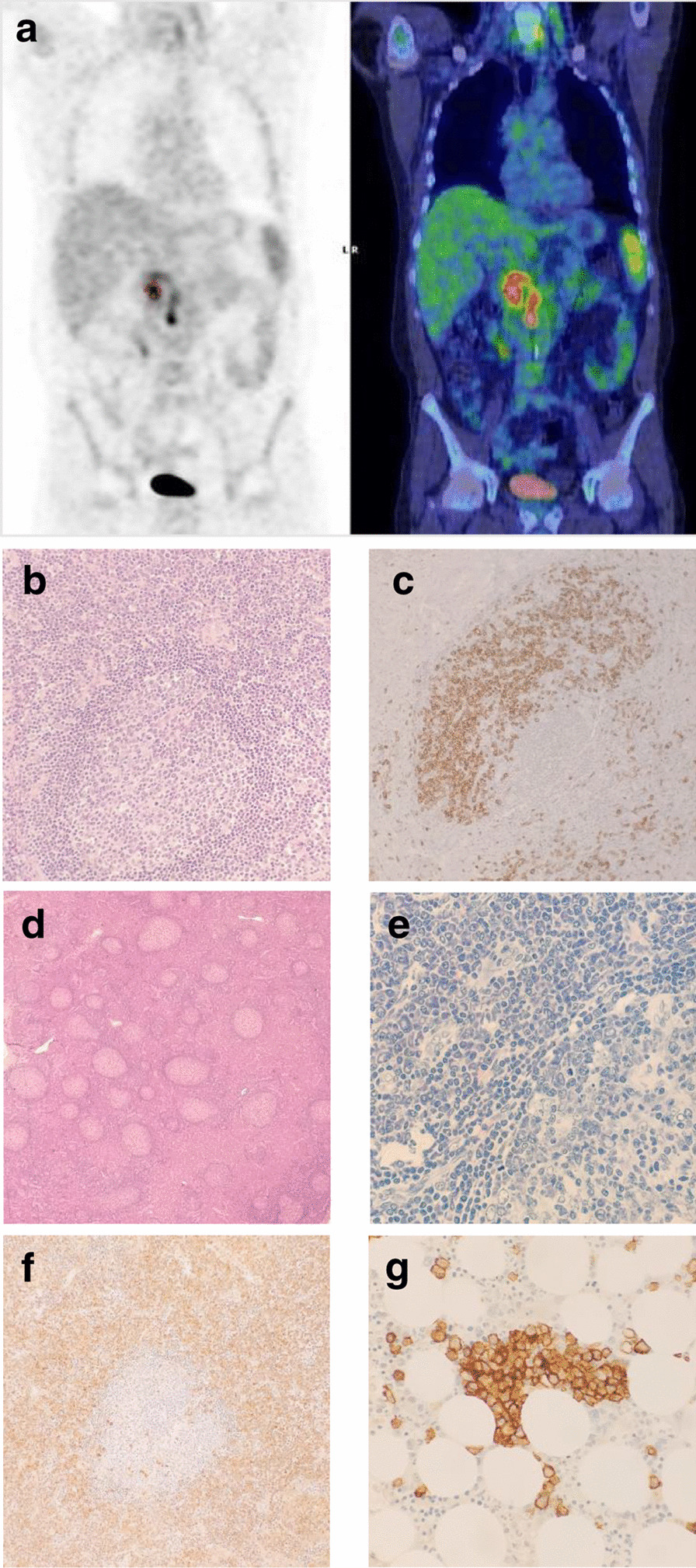
Imaging and pathological findings of iMCD case 1 and case 2. Case 1 CT/PET-scan examination: contrast-enhancing peri-pancreatic and retrocaval adenopathies (**a**); Case 1 formalin-fixed paraffin-embedded (FFPE) lymph node: follicular regression (hematoxylin and eosin [H&E] staining, **b**) and plasmacytosis (CD138 staining, **c**). Case 2 FFPE lymph node (H&E staining, **d**): germinal center fragmentation (Giemsa staining, **e**); plasmacytosis with light chains impaired ratio (k*appa* staining, **f**). Case 2 bone marrow biopsy showing reactive plasmacytosis (CD138 staining, **g**). PET: computed tomography/positron emission tomography; FFPE tissue: Formalin-Fixed Paraffin-Embedded tissue; iMCD: Idiopathic multicentric Castleman disease; H&E: Hematoxylin and eosin

The first case is a 50-years-old Caucasian male hospitalized for Fever of Unknown Origin (FUO) persisting for one month. He daily presented fever till 39.5 °C, associated with nocturnal diaphoresis, asthenia and chills. His initial blood tests showed mild anemia (11 g/dL), normal platelet count (250 k/mL) and leukocytosis (12800 /mL) with neutrophilia and monocytosis. Inflammation markers were elevated (C-reactive protein, CRP, 132 mg/L, erythrocyte sedimentation rate, ESR, 89 mm/h) and slight hypoalbuminemia was found (3.4 g/dL). Blood culture and serological tests excluded the most frequent infections. A whole-body computed tomography (CT) showed small, contrast-enhancing, peri-pancreatic (short axis diameter 10 mm) and retrocaval adenopathies (maximum size 16 x 11 mm), with a maximum fluorodeoxyglucose (FDG) standard uptake value (SUVmax) of 7.1 at positron emission tomography (PET) scan (Fig. 1a). Bone marrow biopsy showed non-necrotizing epithelioid granulomas, as well as an excess of interstitial and perivascular polyclonal plasma cells (about 15%). To address diagnosis, retrocaval lymph nodes were surgically resected. The pathological examination indicated the presence of vascular plasma cells proliferation and expansion along with focal aspects of follicular regression (Fig. 1b, c), concluding for a compatible diagnosis of “HHV-8/HIV negative iMCD, of uncertain histopathological classification”, thus neither clearly attributable to the Hyalino Vascular, nor Plasmacytic nor Mixed subtype. IL-6 resulted elevated (76 ng/mL). According to consensus diagnostic criteria (2), a diagnosis of iMCD was established. Furthermore, the iMCD was classified as *nonsevere *(1)*,* since patient did not present compromised performance status, renal dysfunction, anasarca, severe anemia or pulmonary involvement (1). Consequently, high-dose steroid therapy was administered for one week (prednisone 1 mg/Kg die) and *Siltuximab* (11 mg/kg every 3 weeks) was started, with rapid improvement of systemic symptoms and laboratory parameters. Computed tomography (CT) scan performed after 6 months of anti-IL-6 therapy indicated a complete regression of all previously reported adenopathies. As we report, patient is still receiving *Siltuximab* every 3 weeks maintaining a good clinical response.

## Case n. 2 presentation

The second case is a 49-years-old Caucasian, non-smoker, male acceded to hospital for systemic lymphadenopathy. Initially, blood tests showed increase of erythrocyte sedimentation rate (ESR, 95 mm/h) and hypergammaglobulinemia (IgG > 3000 mg/dL). Medical history was not relevant. Serological tests excluded HIV, Hepatitis C and Epstein-Barr virus. To direct diagnosis, mandibular-angle lymph node was biopsied. Pathological examination (Fig. 1d, f) showed germinal centres fragmentation, plasmacytosis (cytoplasmatic light chains *kappa* to *lambda* ratio 5:1) and HHV-8 negativity, suggesting a diagnosis compatible with HHV-8/HIV negative iMCD, plasmacytic variant. Given the absence of systemic symptoms and organ disfunction, the iMCD was classified as *nonsevere* (1) and the *watch and wait* (*W&W*) strategy was adopted.

After three-years of *W&W*, low-grade fever, malaise, asthenia and severe, diffuse, arthromyalgia occurred. Blood tests showed mild anemia (Hb 12.8 g/dL), normal platelet count (408k /ml), normal lactate dehydrogenase (221 mU/ml), albumin in range (3.7 g/dL), CRP increase (25 mg/L) and hypergammaglobulinemia (IgG 3412 mg/dL). Bone marrow biopsy pathological examination showed trilinear hyperplasia, reactive plasmacytosis (Fig. 1g) and mild excess of cytotoxic lymphocytes. The whole-body computed tomography scan indicated the presence of multiple supra-diaphragmatic and infra-diaphragmatic adenopathies (respectively maximum size, 26 mm × 10 mm and 25 × 20 mm). As next step, PET-scan (also recommended by rheumatologist in order to exclude vasculitis) was performed, confirming a moderate FDG SUV increase in mediastinal, axillary, retro-pectoral lymph nodes (maximum SUV 3.5). IL-6 resulted strongly increased (441 ng/mL). At that time *Siltuximab* was not yet approved for iMCD treatment in Italy. For this reason, in order to control symptoms, after steroid-based therapy failure patient received 4 cycles of *Rituximab *(375 mg/mq) and *Etoposide *(150 mg/m^2^) administered every week, without significant response. Therefore, 3 cycles of *Siltuximab *11 mg/Kg were administered every 3 weeks (in the context of named patient program (3))*,* but neither clinical nor laboratory improvement was observed. For this reason, after one month, a twice-weekly *Tocilizumab 8 mg/Kg* administration was performed for 16 months, but patient showed only mild laboratory improvement and no regression of symptoms nor of adenopathies was observed. Afterwards, *Intravenous immunoglobulin* (IVIG) 25 g/die infusion for 5 days was attempted without results, as well as a further therapeutic effort with Cyclosporine (CsA, target 100–200 mcg/L) associated to *Tocilizumab* for six months. Since persistence of symptoms, firstly *Sirolimus *(target 12–20 ng/ml) for 5 months, then the anti-Interleukin-1 (anti-IL-1) *Anakinra* at a dose of 100 mg/die for 2 months, and then *Siltuximab *11 mg/Kg again for 3 months were administered, but no improvement emerged. As we report, inflammatory syndrome and arthromyalgia are still not controlled. For this reason, since a recent lymph node biopsy confirmed iMCD diagnosis, patient has received thalidomide, cyclophosphamide and prednisone association therapy (4). Despite this, after 5 months of treatment, the association therapy was discontinued since no clinical improvement was observed.

## Discussion and conclusions

The two previously reported cases coming from our hospital real-life are coherent with the evidences that IL-6 blockade is safe and, at least in 30–40% of iMCD patients, is effective (5). Conversely, despite considerable progress made in iMCD knowledge in recent years, patients not responding to IL-6 blockade often lack an effective therapy. Hopely, for IL-6 blockade refractory patients, therapeutic alternatives could be represented by Sirolimus, targeting PI3K/AKT/mTOR signaling (NCT03933904 trial) (6) or by associations of conventional drugs such as thalidomide, cyclophosphamide and prednisone (4). In conclusion, *Siltuximab* is the first on label drug for iMCD frontline treatment; despite being effective and very well manageable, further efforts are required to treat that considerable part of patients who still do not respond to it.

## Data Availability

Not applicable.

## Abbreviations

CD: Castleman disease
iMCD: Idiopathic multicentric Castleman disease
IL-6: Interleukin-6
FUO: Fever of Unknown Origin
CRP: C-reactive protein
ESR: Erythrocyte sedimentation rate
CT: Computed tomography
HIV: Human immunodeficiency virus
HHV-8: Human herpesvirus 8
FDG: Fluorodeoxyglucose
SUV: Standard uptake value
PET: Positron emission tomography
W&W: Watch and wait
IVIG: Intravenous immunoglobulin
CsA: Cyclosporine A
IL-1: Interleukin-1
